# Optimization and scale-up of α-amylase production by *Aspergillus oryzae* using solid-state fermentation of edible oil cakes

**DOI:** 10.1186/s12896-021-00686-7

**Published:** 2021-05-04

**Authors:** M. Balakrishnan, G. Jeevarathinam, S. Kiran Santhosh Kumar, Iniyakumar Muniraj, Sivakumar Uthandi

**Affiliations:** 1 Department of Food Process Engineering, Tamil Nadu Agricultural University, Coimbatore, Tamil Nadu 641 003 India; 2 Biocatalysts Laboratory, Department of Agricultural Microbiology, Tamil Nadu Agricultural University, Coimbatore, Tamil Nadu 641 003 India

**Keywords:** α-Amylase, Solid-state fermentation, *Aspergillus oryzae*, Edible oil cake, Pilot-scale fermenter

## Abstract

**Background:**

Amylases produced by fungi during solid-state fermentation are the most widely used commercial enzymes to meet the ever-increasing demands of the global enzyme market. The use of low-cost substrates to curtail the production cost and reuse solid wastes are seen as viable options for the commercial production of many enzymes. Applications of α-amylases in food, feed, and industrial sectors have increased over the years. Additionally, the demand for processed and ready-to-eat food has increased because of the rapid growth of food-processing industries in developing economies. These factors significantly contribute to the global enzyme market. It is estimated that by the end of 2024, the global α-amylase market would reach USD 320.1 million (Grand View Research Inc., 2016). We produced α-amylase using *Aspergillus oryzae* and low-cost substrates obtained from edible oil cake, such as groundnut oil cake (GOC), coconut oil cake (COC), sesame oil cake (SOC) by solid-state fermentation. We cultivated the fungus using these nutrient-rich substrates to produce the enzyme. The enzyme was extracted, partially purified, and tested for pH and temperature stability. The effect of pH, incubation period and temperature on α-amylase production using *A. oryzae* was optimized. Box–Behnken design (BBD) of response surface methodology (RSM) was used to optimize and determine the effects of all process parameters on α-amylase production. The overall cost economics of α-amylase production using a pilot-scale fermenter was also studied.

**Results:**

The substrate optimization for α-amylase production by the Box**–**Behnken design of RSM showed GOC as the most suitable substrate for *A. oryzae*, as evident from its maximum α-amylase production of 9868.12 U/gds. Further optimization of process parameters showed that the initial moisture content of 64%, pH of 4.5, incubation period of 108 h, and temperature of 32.5 °C are optimum conditions for α-amylase production. The production increased by 11.4% (10,994.74 U/gds) by up-scaling and using optimized conditions in a pilot-scale fermenter. The partially purified α-amylase exhibited maximum stability at a pH of 6.0 and a temperature of 55 °C. The overall cost economic studies showed that the partially purified α-amylase could be produced at the rate of Rs. 622/L.

**Conclusions:**

The process parameters for enhanced α-amylase secretion were analyzed using 3D contour plots by RSM, which showed that contour lines were more oriented toward incubation temperature and pH, having a significant effect (*p <* 0.05) on the α-amylase activity. The optimized parameters were subsequently employed in a 600 L-pilot-scale fermenter for the α-amylase production. The substrates were rich in nutrients, and supplementation of nutrients was not required. Thus, we have suggested an economically viable process of α-amylase production using a pilot-scale fermenter.

## Background

Amylases, identified in the eighteenth century, are found in animals, plants, insects, and microorganisms [1]. They belong to the family of GH13, with eight subfamilies known for hydrolyzing starch into glucose, maltose, and maltodextrins [2]. Amylases include three groups of enzymes, namely, α-amylases, β-amylases, and glucoamylase. Among them, α-amylases have essential applications in a vast number of industrial processes such as food, fermentation, textile, paper, detergent, and pharmaceutical industries, owing to their potential thermal and pH stability [3].

Commercial production of α-amylases using microorganisms such as fungi and bacteria represents around 30% of the world market on enzymes [1]. Fungi are more potential as α-amylase producers than bacteria. Furthermore, it is a suitable candidate for the industrial production of amylases because of the advantages of growth using low-cost renewable substrates, ease of enzyme extraction, broader stability to pH and temperature, and less requirement of a cofactor [4]. Two fermentation methods, such as solid-state fermentation (SSF) and submerged fermentation (SmF), are being employed for α-amylase production. SSF is preferred over SmF, considering the amount of water requirement, enzyme yield, and energy utilization. *Aspergillus* is capable of secreting several extracellular enzymes, including amylases using renewable substrates. Its exploitation for industrial applications is favored because of its high enzyme secreting potential [4].

The primary cost for the production of industrial enzymes is high and is mainly required for of media development. To be economically potential, the use of low-cost carbon sources, and the addition of other substrates, elements, and cofactors to the media formulation, are considered essential for enhancing enzyme production [5]. Valorization of low-cost substrates for the production of high-value products is gaining momentum as bio-based products fetch a price in the market with an added advantage of simultaneous reduction of waste generated [3]. Oil cakes are the byproducts obtained after oil extraction and are commonly used in animal feed, especially for ruminants [6]. Edible oil cakes have high nutritional value, especially protein content ranging from 15 to 50% [7]. Moreover, rich fiber and polysaccharide content with less starch content makes the oil cakes suitable for valorization. Therefore, oil meals/oil cakes such as coconut oil cake, sesame oil cake, cottonseed cake, groundnut oil cake, palm kernel cake, and olive oil cake are considered as potential substrates for enzyme production.

Oil cakes are used as substrates for the production of glucoamylase using *A. niger* [8], phytase using *Rhizopus oligosporus* [9], and lipase using *Candida rugose* [10]. SSF is primarily employed to produce enzymes using low-cost substrates at the industrial level. Industrial-scale fermentation is performed in tray reactors and drum-type fermenters. However, a simple, cost-effective fermenter is the need of the hour.

In addition, process parameter optimization to increase enzyme production using statistical approaches is a prerequisite for pilot or industrial production [11, 12]. Box–Behnken experimental design of response surface methodology (RSM) is an important statistical tool for screening or optimizing the process parameters. The production efficiency of α-amylase using fungi is mainly characterized by its morphology and metabolic potential [13]. While using low-cost substrates and SSF, the high yield of amylase can be achieved by excessive fungal mycelium growth. Moreover, physico-chemical parameters such as pH, temperature, incubation period, carbon sources, and supplements, including inducers and surfactants, play a significant role in obtaining the maximum yield. Most studies indicate that combinational parameters influence the enzyme yield. In this study, the effects of pH, incubation period, and temperature on α-amylase production using *A. oryzae* were analyzed, and the process was optimized by BBD.

The α-amylases production using *A. oryzae* has been studied for SSF using spent brewing grains [14], combined substrates [15], rice husk and bran, wheat bran [16], peanut oil cakes [17], soybean husk, and flour mill [18]. The optimization of process parameters by RSM using spent brewing grains [14] and rice bran [19] has been studied but not using groundnut, coconut, and sesame oil cakes. In the present study, we aimed to use GOC, COC and SOC for SSF to maximize the α-amylase production by *A. oryzae* and to optimize various process parameters by RSM. With these optimized SSF process conditions, pilot-scale production of α-amylase was also attempted.

## Results

### C:N ratio of oil cakes

The total carbon and total nitrogen analysis of three oil cakes showed that the total carbon was the highest in GOC (24%), followed by COC (20%), and SOC (18%). The percentage of nitrogen in all the substrates was 1%.

### Optimization of α-amylase production

The level and range of variables considered to optimize the process parameters for the maximum yield of α-amylase are shown in Table 1. Each replicated value of treatment was considered as a dependent variable or response, i.e., α-amylase activity (U/gds).

**Table 1:** Levels of independent variables using the Box–Behnken design for α-amylase production

Factor	Units	Levels	Range	
			Low	High
pH (A)	–	3	3	6
Incubation temperature (B)	°C	3	25	40
Incubation Period (C)	(h)	3	72	144

### Response surface methodology for α-amylase production using GOC, COC, and SOC as substrates

In this model, GOC showed a pronounced effect on α-amylase production using *A. oryzae.* A positive correlation between all the process parameters and α-amylase production was observed and was the maximum for GOC as the substrate, followed by COC and SOC (Table 2). Multiple regression analysis was performed to analyze the data, and quadratic equations obtained for all the substrates are as follows:

**Table 2:** Design matrix and response for the selected three variables in the Box–Behnken design for α-amylase production using groundnut, coconut, and sesame oil cake as substrates

Run	pH (A)	Incubation temperature (B) (°***C***)	Incubation period (h)	α-Amylase activity (U*/gds)		
				GOC	COC	SOC
1	3	25.0	108	2327.00	1775.70	1423.70
2	6	25.0	108	4549.00	3112.50	2371.85
3	3	40.0	108	1586.60	1086.27	720.00
4	6	40.0	108	1568.00	1059.67	985.24
5	3	32.5	72	6031.00	3068.00	2660.74
6	6	32.5	72	8519.20	2327.40	1475.56
7	3	32.5	144	5290.30	3512.59	2934.81
8	6	32.5	144	6549.00	2075.50	1475.56
9	4.5	25.0	72	5305.00	2238.50	2194.07
10	4.5	40.0	72	2102.50	1122.86	850.00
11	4.5	25.0	144	3912.00	2148.00	2031.10
12	4.5	40.0	144	1898.00	1102.60	896.00
13	4.5	32.5	108	9868.12	4031.10	3068.15
14	4.5	32.5	108	9868.00	4031.10	3068.15
15	4.5	32.5	108	9864.50	4031.10	3068.15
16	4.5	32.5	108	9868.00	4031.10	3068.15
17	4.5	32.5	108	9868.00	4031.10	3068.15

For GOC

1 $$ {R}_1=9867.32+743.79\times A-1117.24\times B-538.55\times C-2033\times {A}^2-5326.34\times {B}^2-1236.61\times {C}^2-540.15\times A\times B-307.38\times A\times C+297.12\times B\times C $$

For COC

2 $$ {R}_2=4030.76+145.19\times A-481.47\times B-4.30\times C-828.76\times {A}^2-1950.72\times {B}^2-456.13\times {C}^2-628.82\times A\times B-174.12\times A\times C+1152\times B\times C $$

For SOC

3 $$ {R}_3=55.38-0.78\times A-7.60\times B-0.72\times C-4.74\times {A}^2-14.47\times {B}^2-3.20\times {C}^2-1.60\times A\times B-2.43\times A\times C+0.69\times B\times C $$

where R_1,_ R_2_, and R_3_ are the enzyme activities (U/gds) for GOC, COC, and SOC, respectively, A is the pH, B is the incubation temperature (°C), and C is the incubation period (h).

The efficiency of the designed model was tested, and Fisher’s statistical analysis was performed to determine the significant parameters. The results showed (Table 3) that the model design was significant (*p* < 0.05) with an F-value of 181.73 for GOC, 10.10 for COC, and 11.17 for SOC. The R^2^ values for GOC, COC, and SOC were 0.99, 0.92, and 0.89, respectively, indicating that the multiple regression coefficients were close to 1, resulting in a better correlation. The values of adjusted R^2^ (0.98, 0.83, and 0.77) and predicted R^2^ (0.93, 0.77, and 0.67) for GOC, COC, and SOC, respectively, confirmed the significance of the model. The *p-*value determines the significance between coefficients and also helps in estimating the interaction between the variables. A, B, C, A^2^, B^2^, C^2^, AB, and AC were significant variables for GOC; B, A^2^, B^2^, and C^2^ were significant variables for COC; and B, C, A^2^, B^2^, and C^2^ were significant variables for SOC. The variables having *p*-values greater than 0.10 were insignificant. This showed that pH, incubation temperature (°C), and incubation period (h) exert a strong effect on the α-amylase production using *A. oryzae* with GOC as a substrate.

**Table 3:** ANOVA for quadratic model for α-amylase production in the Box–Behnken design using groundnut (GOC), coconut (COC), and sesame (SOC) oil cakes as substrates

Sources	df	GOC	COC	SOC
Model	9	181.73**	10.10**	11.27**
A	1	42.38**	0.42^NS^	1.88^NS^
B	1	95.39**	13.99**	20.55**
C	1	22.16**	8.348×10^−3NS^	6.95*
A^2^	1	164.24**	6.52*	17.49**
B^2^	1	1135.68**	53.09**	30.97**
C^2^	1	60.26**	9.06**	12.28**
AB	1	11.99**	2.07*	0.92^NS^
AC	1	3.61**	0.54^NS^	3.54^NS^
BC	1	3.37^NS^	0.014^NS^	0.086^NS^
Residual	7			
Pure error	4			

**p significant at 1% level, and *p significant at 5% level; R^2^, 0.89; adjusted R^2^, 0.77; predicted R^2^, 0.67

A = pH,

B = incubation temperature (°*C*),

C = incubation period (h),

R^2^ = multiple regression coefficient,

df = Degree of freedom,

NS = not significant, and

*p* value = significance of coefficients

### Optimization of process parameters

**Fig. 1:**
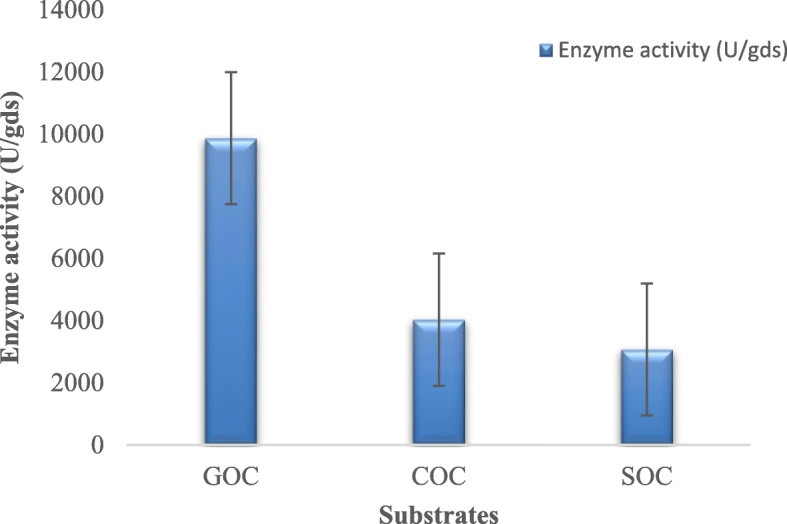
Effect of substrates on enzyme activity

**Fig. 2:**
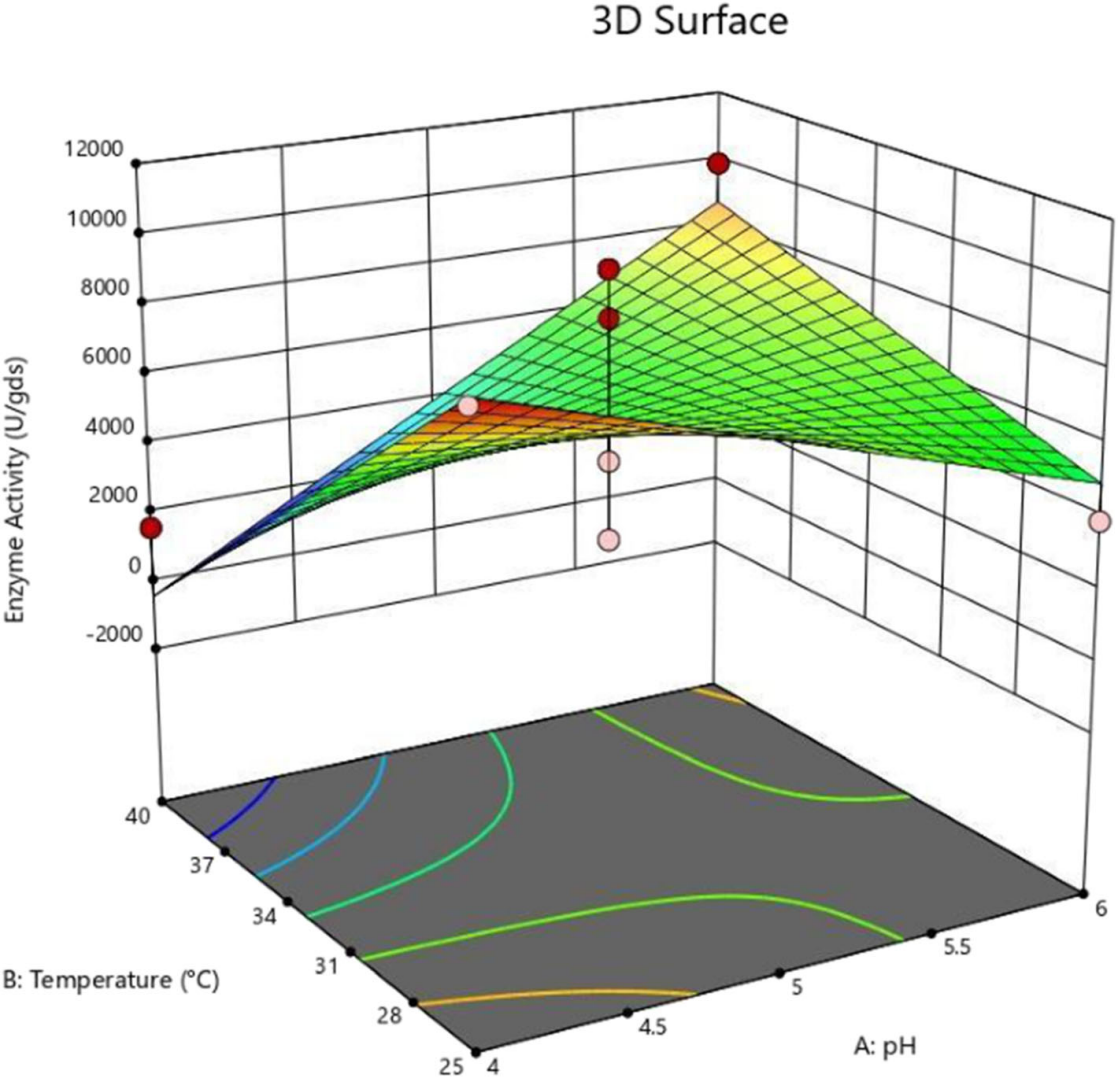
Response surface analysis of process parameters on α-amylase activity using groundnut oil cake as the substrate

**Fig. 3:**
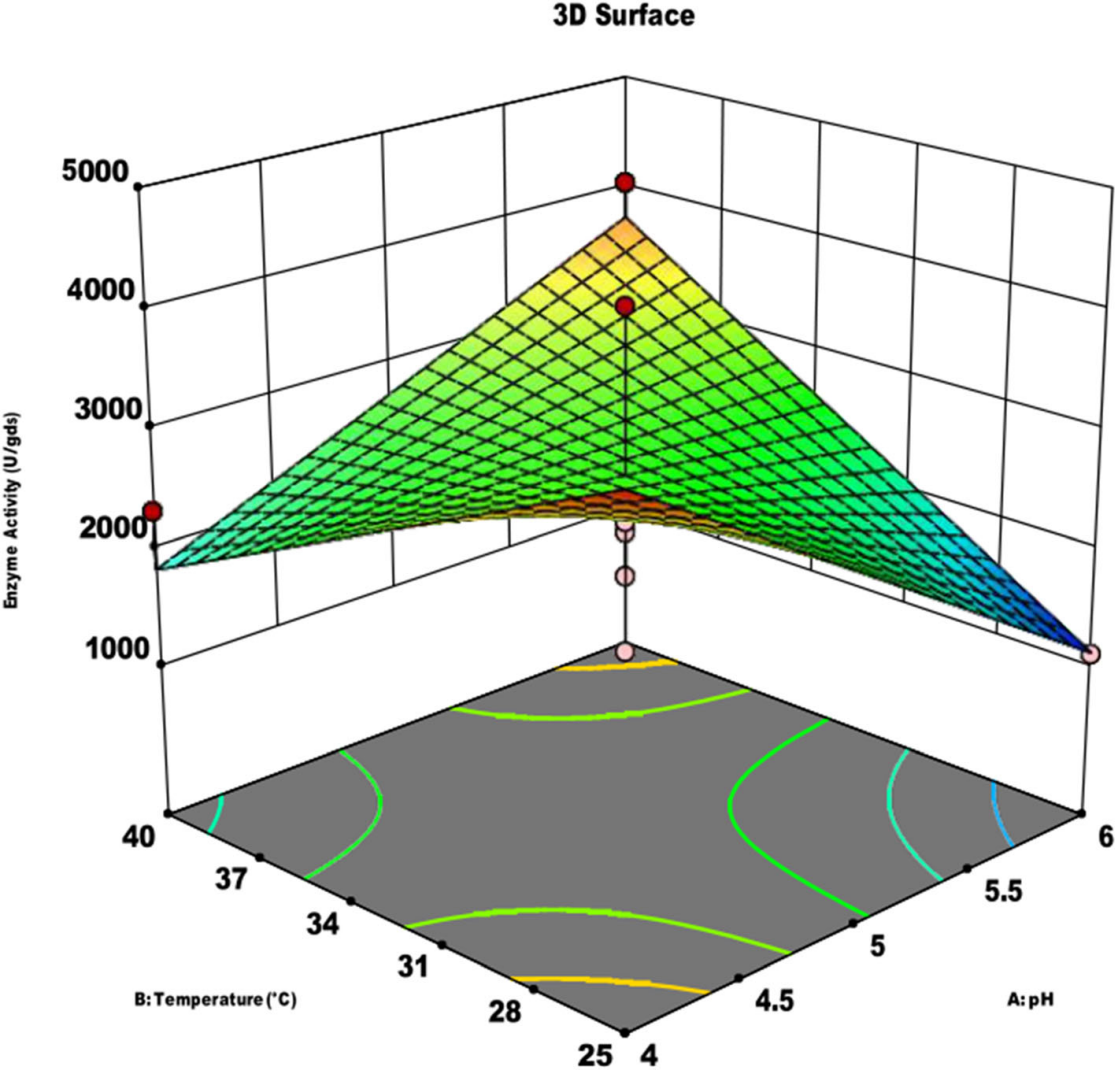
Response surface analysis of process parameters on α-amylase activity using coconut oil cake as the substrate

**Fig. 4:**
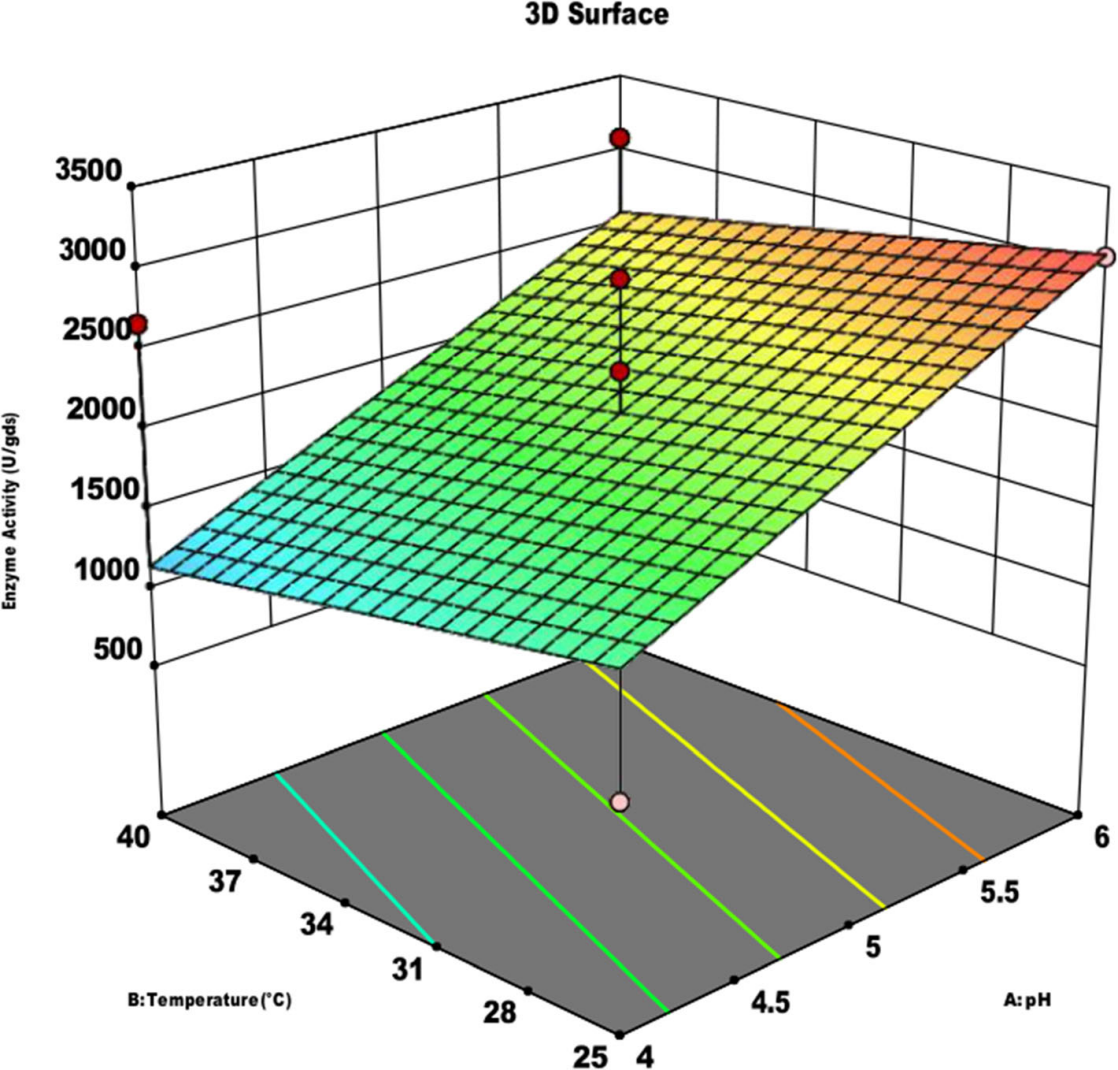
Response surface analysis of process parameters on α-amylase activity using sesame oil cake the substrate

The effect of substrates on the α-amylase activity of *A. oryzae* is shown in Fig. 1. GOC was found to be a suitable substrate for maximum enzyme production of 9868.12 U/gds, followed by 4031.12 U/gds using COC and 3068.15 U/gds using SOC. The effects of pH, incubation temperature, and incubation time on α-amylase activity using the three edible oil cakes are shown in Figs. 2, 3 and 4. The maximum amylase production was observed at 32.5 °C, pH 4.5, an incubation period of 108 h using all the three oil cakes (Figs. 2, 3 and 4). An increase in temperature to above 32.5 °C decreased enzyme production.

Interactions between the process parameters were also studied. While analyzing the effect of process parameters using a three-dimensional (3)-contour plot (Figs. 2, 3 and 4), it was found that temperature was a more significant parameter affecting the enzyme production than pH, as the central contour points were heading toward the temperature level for all the three substrates. In the interaction between pH and incubation period for α-amylase production, contour lines were more oriented toward the pH. Hence, we concluded that pH had a more significant effect on α-amylase production than the incubation period.

### Comparison between pre- and post-optimization

A preliminary study was performed to estimate the amount of α-amylase produced using GOC, COC, and SOC under various process parameters such as pH, incubation temperature, and incubation period. The increase in α-amylase activity (6281.47 U/gds) was observed at pH 6, incubation temperature of 35 °C, and an incubation period of 145 h and a decrease in the activity (4657.93 U/gds) was observed at pH 3, an incubation temperature of 25 °C, and an incubation period of 72 h. Based on the preliminary study results, ranges of process parameters were optimized and used for the Box**–**Behnken design for post-optimization to achieve effective α-amylase production using the oil cakes. The post-optimization showed a maximum α-amylase production of 9868.12 U/gds with pH 4.5, a temperature of 32.5 °C, and an incubation period of 108 h. This combination of process parameters yielded higher α-amylase production than other combinations, resulting in the Box**–**Behnken design.

### Production of α-amylase in a pilot-scale solid-state fermenter and cost economics

RSM-optimized parameters were employed to enhance the α-amylase production in a pilot-scale solid-state fermenter. Under the optimized parameters of a pH of 4.5, an incubation temperature of 32.5 °C, and an incubation period of 108 h, *A. oryzae* produced α-amylase to a tune of 10,994.74 U/gds using GOC. The overall cost of production was estimated with the total fixed cost and variable cost of Rs. 44,306/− and Rs. 4,22,819.32/−, respectively annually. The overall production cost for the production of α-amylase annually was estimated to be Rs. 622/L of amylase.

### Partial purification and stability of α-amylase

**Fig. 5:**
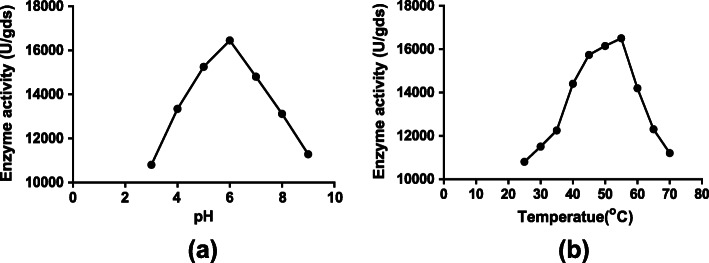
Optimization of (**a**) pH and (**b**) temperature stability of partially purified α-amylase

Ammonium sulfate precipitation of α-amylase provided one-fold purification with an activity of 10,800 U/gds, and the partially purified protein was used for studying pH and temperature stability. The results of pH and thermal stability of α-amylase shown in Fig. 5 indicated that the maximum stability was found at pH 6, whereas pH below and above this value was detrimental. The maximum thermal stability was recorded at 55 °C for 10 min; a further increase in temperature resulted in a decrease in α-amylase activity.

## Discussion

In the present study, RSM-mediated optimization of process parameters for α-amylase production was performed using edible oil cakes with SSF. Previous studies on the optimization of α-amylase production have considered factors such as modifying nutritional parameters (C:N ratio, supplements, and other nutrients), physiological conditions (temperature, pH, inoculum load, and incubation time), the superiority of organisms (fungi and bacteria), and the method of fermentation (SSF and SmF) [1, 12]. One factor at a time (OFAT) optimization was predominantly used to optimize α-amylase production [12, 20–25]. Because of the tedious nature of OFAT, statistical optimization tools are preferred for optimization studies, especially for α-amylase production, since the past decade [24, 25]. Statistical tools provide an advantage of simultaneous optimization compared with OFAT with a range of design variables suited for each optimization program. In the present study, RSM optimization achieved an α-amylase production of 9868.12 U/gds using GOC. The use of low-cost substrates for α-amylase production is a common practice to reduce the cost. We found COC as one of the suitable substrate for α-amylase production using *A. oryzae*. Ramachandran et al. [22] followed the OFAT approach using COC and supplements and recorded a yield of 3388 U/gds of α-amylase [22].

Similarly, spent brewing waste was used as a cheap substrate, and Plackett–Burman based RSM was used for the optimization of the α-amylase production by *A. oryzae* NRRL 1808 (=ATCC 12892). Amylase production of 4519 U/gds has been reported to be achieved in 3 days under optimum conditions [26]. *Fusarium soloni* was employed for the production of α-amylase using mango stone kernel. The α-amylase yield of 0.889 U/gds was achieved with 5% (w/v) substrate concentration at pH 4.0 and temperature of 30 °C in 9 days [27]. *A. terreus* NCFT4269.10 was used for α-amylase production under both liquid static and solid substrate fermentation using pearl millet as a substrate. Pear millet substrate yielded a maximum amylase yield of 19.19 ± 0.9 U/gds by SSF. The increased yield was attributed to additional substrates added to pearl millet such as magnesium sulfate (1 mM), gibberellic acid (0.025% [w/v]), and vitamin E (30 mg/100 mL [w/v]). The optimized process conditions were pH of 7.0 and incubation temperature of 30 °C for 96 h [4]. *A. oryzae* MTCC-8624 was used for α-amylase production using rice bran with SmF. The experimental parameters were optimized using a Box–Behnken design. The optimum conditions were found to be 16.89 U/mL at a pH of 4.7, the temperature of 36.5 °C, fermentation time of 66.6 h, inoculum concentration of 5.7%, and substrate concentration of 18.9% [28]. The results are in accordance with our findings and confirmed our findings. After comparing the α-amylase yields of all the studies, irrespective of the method of optimization, we found that the yield obtained in the present study was higher. Reasons for the high yield can be the nature of the substrates and their nutritive values, the secretory potential of fungi, and the optimization conditions of RSM. Moreover, several studies had supplemented the substrate with either carbon source, nitrogen source, or other additives to improve the yield of α-amylase. However, no supplementations were incorporated in the present study. This encouraged and supported the RSM method of optimization for yielding a high amount of the enzyme, especially with *A. oryzae* using edible oil cakes. The parameters optimized in this study were employed for pilot-scale fermentation for the maximal production of α-amylase, and the results showed a 1% increased yield. Pilot-scale optimization is a prerequisite technique for the industrial production of enzymes. A GROWTEK bioreactor was designed to overcome the limitations of the tray-type solid state fermenter. In another study, *A. oryzae* was cultivated in SSF and attained a maximum yield of 12,899 U/gds of amylase at 30 °C after 66 h of incubation. The yield of SSF was further increased to 15,480 U/gds when supplemented with 0.1% Triton X-100 [29]. However, in the present study, less increase in the α-amylase yield in a pilot-scale fermenter might be because of the absence of supplements. Partially purified α-amylase showed thermal and pH stability, which can potentially be used in the detergent industry and for the biodegradation of food waste [3]. Many studies have been performed to demonstrate improved thermal and pH stability of enzymes having industrial applications [2, 30–32].

## Conclusions

The α-amylase production using *A. oryzae* under SSF was performed to optimize parameters in the presence of different edible low-cost deoiled cakes as substrates by using RSM. The process parameters such as incubation temperature, pH, and incubation period had a significant effect (*p* < 0.05) on the α-amylase activity. Under the optimum process conditions, the maximum α-amylase activity of 9868.12 U/gds was attained when GOC was used as a substrate at a pH of 4.5, incubation temperature of 32.5 °C, and an incubation time of 108 h. The partially purified α-amylase exhibited the maximum stability at a pH of 6.0 and a temperature of 55 °C. The pilot-scale production of α-amylase using a solid-state fermenter of capacity 600 L was attempted using GOC as a substrate at a pH of 4.5, incubation temperature of 32.5 °C, and incubation period of 108 h and the enzyme activity of about 10,994.74 U/gds was obtained. Therefore, the optimized process can be used for commercial enzyme production. The improved thermal and pH stability of the enzyme could be an added advantage for many industrial applications.

## Methods

### Microorganism, culture conditions, and substrates

The fungal strain *viz*., *A. oryzae* (MTCC 3107) was obtained from the Microbial Type Culture Collection (MTCC), Chandigarh. *A. flavus* and *A. oryzae* cultures were maintained on yeast extract malt extract agar and Czapek Dox agar, respectively. Potato–dextrose agar was also used for the growth and maintenance of the cultures. The cultures were grown at 30 °C for seven days and then stored at 4 °C. The powdered edible oil cakes, namely groundnut, coconut, and sesame oil cakes were selected as substrates. GOC and COC were purchased from M/s. Savithri Oil Mill, Coimbatore, and SOC were purchased from M/s. SSD Oil Mill Company, Coimbatore. The composition of oil cakes such as total carbon and nitrogen content was estimated using the total carbon analyzer Leco CS300 and by the Kjeldahl method, respectively [33]. Dry weights of the substrates were estimated by drying them in a hot air oven (Hasthas Make model D1005) at 105 °C for 24 h until a constant weight. The substrates were powdered and sieved using standard sieves to eliminate the foreign materials and stored in aseptic conditions for further experiments.

### Inoculum preparation and solid-state fermentation

Spores of 7-day-old fungal cultures were scrapped using an inoculation loop and aseptically transferred to sterile distilled water containing 0.1% Tween-80. Exactly 1 mL (1 × 10^7^ mL^− 1^ spores) of spore suspension was used as inoculum for the entire fermentation. About 5 g of dry oil cake was taken into a 250-mL Erlenmeyer flask, containing 2 mL of mineral salts solution containing 2 g of potassium dihydrogen phosphate (KH_2_PO_4_), 5 g of ammonium nitrate (NH_4_NO_3_), 1 g of sodium chloride (NaCl), and 1 g of magnesium dihydrogen sulfate (MgSO_4_.7H_2_O) in a liter of distilled water to adjust the required moisture level. All the contents were mixed, autoclaved at 121 °C for 20 min, and cooled. Spore suspensions were inoculated on the sterile solid substrate and incubated in a solid-state fermenter (M/s. Lark Innovative, Chennai) maintained at 37 °C.

### Development of a pilot-scale solid state fermenter

Amylase production was optimized using a designed solid-state fermenter. A pilot-scale solid-state fermenter of 600 L capacity was fabricated at the Department of Food and Agricultural Process Engineering, Tamil Nadu Agricultural University, Coimbatore. A fan was fixed at the center of the chamber to maintain the temperature. A relative humidity (RH) sensor was placed inside the chamber to monitor the RH of the chamber. A compressor and water supply were connected to spray water inside the chamber whenever RH decreased below the desired level. Under optimum conditions, the microorganisms or cells were able to perform their desired functions. Temperature and RH inside the chamber were monitored and controlled using sensors and controllers, respectively. The bioreactor had four trays, an RH sensor, a temperature controller, and a control system networked together. A thermocouple or Pt 100 was incorporated into the pH sensor. The pH electrode working in the range of 0 to 13 was used. When pH change exceeded the set range, 0.1 N HCl or 0.1 N NaOH was added based on the measured value.

### Enzyme extraction and assay conditions

A known quantity of fermented substrate was mixed with double distilled water along with 0.1% Tween 80. The contents were shaken in a rotary shaker and then centrifuged at 7000 g at 4 °C for 10 min. The reaction mixture consisted of 1.25 mL of 1% soluble starch, 0.25 mL of 0.1 M acetate buffer (pH 5.0), 0.25 mL of distilled water, and 0.25 mL of crude enzyme extract. After 10-min incubation at 50 °C, the liberated reducing sugars (glucose equivalents) were estimated using the dinitrosalicylic acid (DNS) method [34]. The intensity of color developed was measured at 540 nm using a Shimadzu UV-160A spectrophotometer [34]. The blank contained 0.5 mL of 0.1 M acetate buffer (pH 5.0), 1.25 mL of 1% starch solution, and 0.25 mL of distilled water. One unit (IU) of α-amylase was defined as the amount of enzyme releasing one μmol glucose equivalent per minute under the assay conditions.

### Enzyme purification

The crude enzyme was saturated up to 50% using ammonium sulfate and incubated at 4 °C overnight for precipitation of proteins. The sample was centrifuged for 15 min (7000 rpm at 4 °C) in a refrigerated centrifuge (ThermoFisher X1, India). The supernatant-containing residues were discarded and the precipitate was used as an enzyme source.

### Effect of pH and temperature on enzyme stability

The effect of pH on enzyme stability was studied by incubating 0.5 mL of the crude enzyme at different pH ranging from 3 to 9 [35], and varying temperatures ranging from 25 to 70 °C [28]. The pH was altered by varying the concentrations of citrate buffer and glycine-NaOH buffer solutions.

### Optimization of process parameters for production of α-amylase using a statistical approach

The production of α-amylase by *A. oryzae* was investigated using the Box–Behnken design. About 17 experimental runs were performed to determine the effect of all process parameters on α-amylase production. The effect of pH (range 3–6) [35] incubation temperature (range 25–40 °C) [28], and incubation time (range 72–120 h) [35] on the production of α-amylase produced by *A. oryzae* using edible oil cake as a substrate was evaluated (Table 1). The BBD was used to determine the levels of significance and interaction between independent variables. The independent variables were studied at three different levels *viz*., low (− 1), medium (0), and high (+ 1). All experiments were performed in triplicate, and the amount of α-amylase produced was considered as a dependent variable or response (Y). The second-order polynomial coefficients were calculated and analyzed using the “Design Expert” (Version 6.0.8, Stat-Ease Inc., Minneapolis, USA) statistical package. Statistical analysis (analysis of variance) of the model was performed. The analysis also included Fisher’s F-test (overall model significance), its associated probability p (F), correlation coefficient (R), and determination coefficient (R^2^) to measure the goodness of fit of the regression model according to Dey et al. [36]. The regression equation to explain the influence of pH, incubation temperature, and incubation period on α-amylase production is given as.

4 $$ R={b}_o+{b}_1{X}_1+{b}_2{X}_2+{b}_3{X}_3+{b}_{11}{X}_1^2+{b}_{22}{X}_2^2+{b}_{33}{X}_3^2+{b}_{12}{X}_1\times {X}_2+{b}_{13}{X}_1\times {X}_3+{b}_{23}{X}_2\times {X}_3 $$

where R is the response calculated by the model; b_o_ is the intercept; b_1_, b_2_, and b_3_ are the linear effects; b_11_, b_22_, and b_33_ are quadratic effects; b_12_, b_13_, and b_23_ are the interaction coefficients; X_1_ is the pH; X_2_ is the incubation temperature (°C); and X_3_ is the incubation period (h).

## Data Availability

All data are included in the manuscript. No separate external data source is required. Any additional information required will be provided by communicating with the corresponding author via the official e-mail: bala_tnau@yahoo.com.

## Abbreviations

ANOVA: Analysis of variance
GOC: Groundnut oil cake
SOC: Sesame oil cake
COC: Coconut oil cake
df: degrees of freedom
DNS: Dinitrosalicylic acid
gds: gram dry substrate
KH_2_PO_4_: Potassium dihydrogen phosphate
RSM: Response surface methodology
SmF: Submerged fermentation
SSF: Solid state fermentation
MTCC: Microbial Type Culture Collection
U: Unit
μ: micro
α: alpha
%: percentage
